# Accuracy of single intravenous access iohexol GFR in children is hampered by marker contamination

**DOI:** 10.1038/s41598-021-02759-1

**Published:** 2021-12-01

**Authors:** Thea Tislevoll Eide, Karl Ove Hufthammer, Atle Brun, Damien Brackman, Einar Svarstad, Camilla Tøndel

**Affiliations:** 1 Renal Research Group, Department of Clinical Medicine, University of Bergen, Bergen, Norway; 2 Centre for Clinical Research, Haukeland University Hospital, Bergen, Norway; 3 Laboratory for Clinical Biochemistry, Haukeland University Hospital, Bergen, Norway; 4 Department of Clinical Science, University of Bergen, Bergen, Norway; 5 Department of Pediatrics, Haukeland University Hospital, 5021 Bergen, Norway

**Keywords:** Medical research, Nephrology

## Abstract

Measurement of glomerular filtration rate (GFR) in children by iohexol injection and blood sampling from the contralateral arm is widely used. A single intravenous access for iohexol injection and subsequent blood sampling has the obvious advantages of being less painful and easier to perform. The purpose of our study was to determine if blood samples drawn from the injection access are feasible and accurate for iohexol GFR (iGFR) measurements. Thirty-one children, median age 10.5 (range 6–17) years, with chronic kidney disease were given a bolus of iohexol followed by extended saline flushing and subsequent venous blood samples collected from the injection access as well as from a cannula in the contralateral arm, the latter serving as the reference method. Paired venous blood samples were collected at four time points (2, 3, 3.5 and 4 h) after the iohexol bolus. Blood sample discarding preceded and saline flushing followed each blood sampling to avoid marker contamination. iGFR based on samples drawn from the injection access at 2 and 3 h showed significantly lower iGFR than measurement from the contralateral arm (p < 0.01). Singlepoint iGFR did not differ significantly after 3–4 repeated procedures of blood discarding and saline flusing (3.5 and 4 h). Despite thorough saline flushing there is still a relatively high risk of falsely low iGFR due to marker contamination in blood samples from the injection site. Hence, blood sampling from a second intravenous access is recommended for routine iohexol GFR measurements in children.

Clinical trial registration: ClinicalTrials.gov, Identifier NCT01092260, https://clinicaltrials.gov/ct2/show/NCT01092260?term=tondel&rank=2.

## Introduction

Renal inulin clearance is considered the gold standard method for measurement of glomerular filtration rate (GFR) in children. This method is, however, complicated, and inulin is not easily available anymore^1–3^. Several exogenous markers have been evaluated, and multipoint pharmacokinetics methods have been adopted as new gold standard procedures for measured GFR (mGFR) using markers like iohexol and ^51^CrEDTA^2–4^. In clinical practice, serum creatinine based calculation of estimated GFR (eGFR) is the most common method for evaluating renal function as it is simple and has low cost, although clear limitations with low accuracy is well known especially in paediatrics due to the correlation to muscle mass^5,6^. Hence, accurate mGFR methods based on external markers is of great importance for children with renal diseases and for children treated with nephrotoxic drugs due to malignant diseases where muscle mass can change considerably during the treatment^7^.

Iohexol plasma clearance was introduced as a method to measure GFR in the 1980s^3,8^, corresponds well to inulin clearance^9–11^ and is increasingly used in clinical practice^16,17^. Major advantages by using iohexol bolus injections for GFR measurement include safety, simplicity, tolerance, stability, low cost and low inter-laboratory variations^3,12–17^.

Most laboratories use blood samples drawn from the contralateral arm to measure iohexol plasma concentration. To eliminate any risk of contamination, guidelines recommend that blood samples should not be taken from the site of injection^18^. However, it is often challenging to gain intravenous access in children. As a consequence, a method allowing injection and blood sampling from a single intravenous access would be highly welcomed and would potentially simplify and facilitate the monitoring of iohexol GFR in children.

Brøchner-Mortensen stated, based on their own unpublished data, no significant contamination of plasma samples drawn from the injection cannula when 15 mL isotonic saline was injected following the administration of the marker ^51^CrEDTA in one of the taps of the cannula and 10 mL saline through the other tap^19^. Stake et al. used this procedure to establish a new single-plasma sample method in children based on injecting iohexol and drawing blood samples from the same cannula^20,21^. Brändström et al. stated also based on unpublished data no difference in GFR values when comparing blood samples drawn from the injection arm to samples from the contralateral arm in 14 adult patients after post-injection flushing of the intravenous access with 30 mL physiologic saline^22^. To our knowledge, there are no published studies comparing the agreement of iohexol plasma concentration sampled from the injection cannula to iohexol plasma concentration in samples from a contralateral venous access.

The purpose of this study was, given extensive post-injection flushing and pre-sampling blood discarding procedures, to determine if blood samples drawn from the same intravenous access as injection of iohexol are feasible and accurate for measurement of the iohexol plasma concentration, compared to samples from the contralateral arm. To validate the agreement between the venous blood samples drawn from both arms, we compared four different time points for each of the methods i.e. pharmacokinetic (PK) sampling after saline flushing and blood discarding one, two, three or four times.

## Results

**Figure 1:**
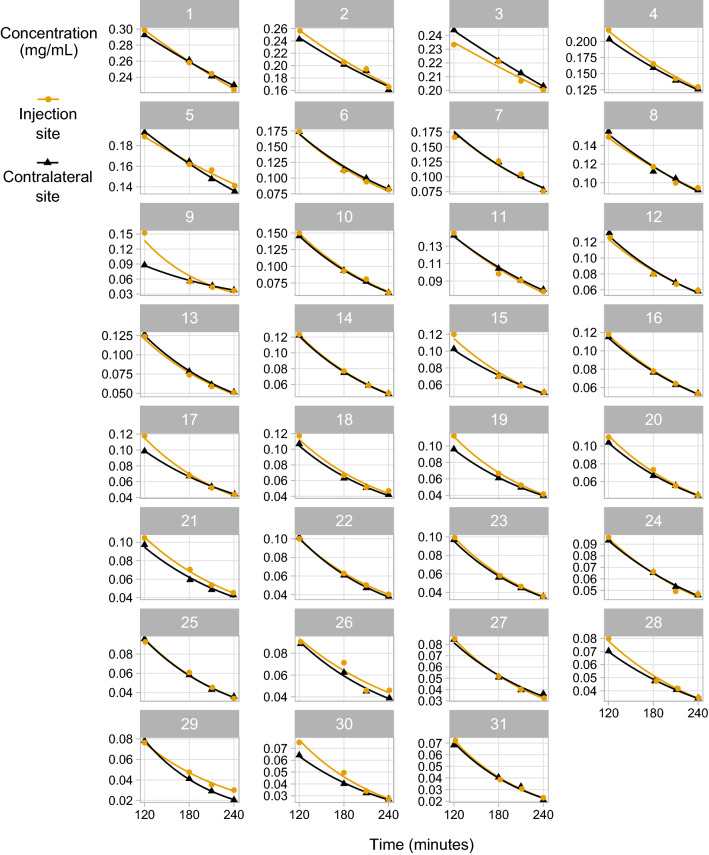
Measured iohexol concentration levels for both arms for the four time points, along with fitted concentration curves based on Jødal–Brøchner-Mortensen’s formula^26,27^. The model showing more similar values at 3.5 and 4 h time points for most of the patients.

Iohexol concentrations for each individual patient at various time points are illustrated in Fig. 1. The discrepancy between the paired measurements is in general larger at 2 h than at the other three time points. Still, some variation is observed also in the samples at 3.5 and 4 h.

**Figure 2:**
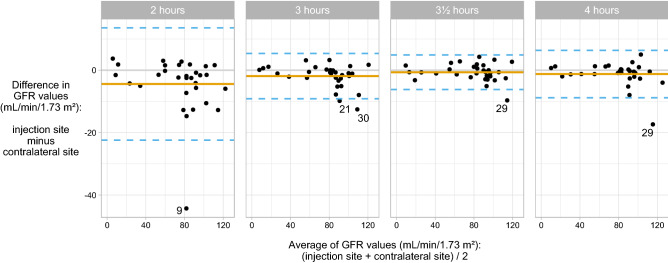
Bland–Altman plots of difference in measured iohexol-GFR (calculated by the Fleming 1-point formula) from samples drawn in parallel from both arms at four time points (2, 3, 3.5 and 4 h): mean bias (solid orange line) and limits of agreements (dashed blue line).

Agreement between calculated iohexol-GFR (iGFR) from paired samples drawn at 2, 3, 3.5 and 4 h using Bland–Altman plots is illustrated in Fig. 2. All the one-sample iGFR were based on the 1-point Fleming formula due to previous shown best performance^23,24^. The results show smaller bias, less variation and fewer outliers in the samples drawn at later time points, suggesting more consistent iGFR for all patients. Calculations of iGFR from the paired samples drawn 2 h after injection showed clear lack of agreement, with unreliable measurements (lower mGFR) from the injection site compared to the reference method. Patient nine is a clear outlier at the 2-h time point, with 43% lower iGFR estimate for the measurement from the ipsilateral arm (60 mL/min/1.73 m^2^) than from the contralateral arm (104 mL/min/1.73 m^2^). For the other time points, the iGFR estimate ranged from 99 to 103 mL/min/1.73 m^2^ for both arms.

**Figure 3:**
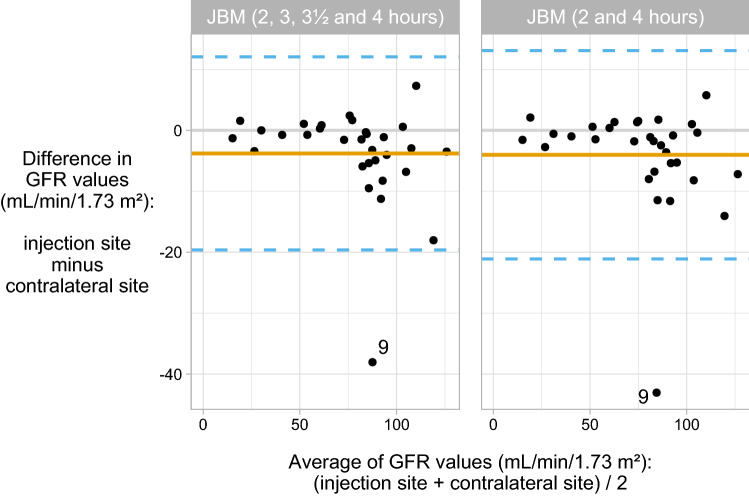
Bland–Altman plots of difference in measured iohexol-GFR (calculated by the JBM 2-point and 4-point formula) from samples drawn in parallel from both arms: mean bias (solid orange line) and limits of agreements (dashed blue line).

Figure 3 shows the corresponding differences when using two or four time points and the JBM formula^26,27^. Measuring GFR using multiple time points gives a more reliable estimate than using one time point, but there is still a notable bias, mainly caused by differences in measured concentration at 2 h.

The performances at four different time points are shown in Table 1. The analysis shows best agreement between the two single-point procedures is found at the 3.5-h time point with the least amount of bias, the tightest limits of agreement and the highest *p*-value (0.24). For measurements at 2 and 3 h, the results with the Fleming formula are statistically significantly different between the samples drawn from the injection cannula and the reference method, suggesting unreliable results due to marker contamination.

**Table 1:** Bland–Altman data comparing mGFR estimates (mL/min/1.73 m^2^) based on samples taken from the ipsilateral and contralateral arm.

Method	Bias	95% CI for bias	LoA	*P*-value*
JBM 4-point (2, 3, 3½ and 4 h)	− 3.8	− 6.7 to − 0.9	− 19.6 to 12.1	0.002
JBM 2-point (2 and 4 h)	− 4.0	− 7.2 to − 0.9	− 21.1 to 13.1	0.002
Fleming 1-point (2 h)	− 4.4	− 7.7 to − 1.1	− 22.4 to 13.6	0.004
Fleming 1-point (3 h)	− 1.9	− 3.2 to − 0.6	− 9.1 to 5.4	0.009
Fleming 1-point (3½ hours)	− 0.7	− 1.7 to 0.4	− 6.2 to 4.9	0.24
Fleming 1-point (4 h)	− 1.2	− 2.6 to 0.2	− 8.8 to 6.4	0.06

*CI* confidence interval, *LoA* limits of agreement, *JBM* Jødal–Brøchner–Mortensen.

*P-value calculated by the Wilcoxon signed rank test (testing symmetry around 0).

Comparisons between the two intravenous accesses for two time points and four time points using the JBM formula are also shown in the same table. There were little overall differences in agreement between two and four time points, but both procedures had poor agreement, with generally lower iGFR estimates from the ipsilateral arm, likely due to both procedures including the 2-h time point.

## Discussion

Poor agreement was found between paired samples from injection site and contralateral arm collected at time point 2 h after injection, with an unacceptable discrepancy in the calculated iohexol-GFR values (Table 1). Likewise, the samples drawn at time point 3 h after injection shows a suboptimal agreement between the test method and reference method, with a risk of underestimating the iGFR. However, the GFR 1-point measurements using the Fleming formula shows a smaller discrepancy between paired iGFR by samples drawn from the cannulae at 3.5 and 4 h after injection. At these time points, the results indicate more agreement between the two sampling methods, with lower bias and better limits of agreement (Table 1, Fig. 2). The reason for better agreement after multiple samplings is likely the increased rinsing of the cannula through movement of the rubber inside the cannula due to repeated procedures with blood discarding and saline injection. This is in agreement with results observed in the study showing less discrepancy between the paired samples collected at 3.5 and 4 h after injection of iohexol. The persistent minor difference is likely due to analytical variation as previous studies show an intra-individual coefficient of variation of 5.6%^28^ and 5.4%^29^ using iohexol clearance.

Due to need of one relatively early sample using the slope methodology^4,25–27^, JBM calculations with both two sampling points and four sampling points resulted in unreliable iGFR-values from the ipsilateral cannula (Table 1, Fig. 3). Both calculation methods included the samples drawn 2 h after injection, which is shown to be unreliable and inaccurate (Fig. 1). Yet, the risk of outliers is an issue in every test. If using a single-point procedure it would therefore be necessary to redo the test if the result seems unreliable, whereas a multi-point GFR procedure would normally be more accurate and have the possibility to remove an outlier reviewing the examination of the elimination curve. However, using blood sampling from the ipsilateral cannula, the risk of contamination of the sample with remnants of marker is the highest at the first samples, i.e. on a time point where it is not possible on the eliminations curve to see that it is an outlier (Fig. 1). In a real-world clinical caring perspective the benefit of single access sometimes may outweigh the methodological inaccuracy. However, if the clinician deem the highest accuracy of GFR of critical importance in specific cases, we recommend a standard GFR method sampling from a contralateral intravenous access.

Endogenous substances, such as creatinine, are widely used to estimate kidney function in clinical practice. However, formulas used to estimate GFR in children often show low accuracy, with studies reporting less than 50% of eGFR based on serum creatinine, cystatin C and/or urea within ± 10% of the reference method^5,6^. Feasible methods to measure GFR using an infused marker with purely renal clearance is therefore of great importance. In our study iohexol clearance has been used for GFR determination and shows good agreement for single access procedure at the time points at 3.5 and 4 h, provided repeated saline flushing and blood discarding at earlier time points.

Previous publications on both children and adults have stated no contamination by using the same cannula for marker injection and blood sampling when flushing the taps with similar amounts of physiologic saline as in our study^19–21^. To our knowledge, this statement has not been confirmed using systematic statistical analysis in any publication. Stake et al. used this method to establish a single plasma sample method with iohexol to estimate GFR^20,21^. In their study, concern about the possible marker contamination of the blood sample using such method was raised^21^. Despite their concern, they still used this method to measure the renal function in the patients. They showed that GFR estimates based on the first sample drawn 1 h after iohexol injection were clearly inaccurate compared to samples collected at later time points, which is consistent with the results for the first sampling point found in our study. The findings from Stake et al. may be explained due to sampling outside the time limits of the formula used for calculation, but it is likely that contamination by the marker itself also may have influenced results^20,21^.

Small remnants of iohexol particles in the cannula used for injection is a likely explanation for the iGFR discrepancies found in our study, even after flushing considerable amounts of physiologic saline through the taps of the cannula. For the third and fourth sampling time points, i.e. at 3.5 and 4 h, however, where blood sampling procedures including blood discarding and saline flushing had already been repeated several times, there was no significant difference between the paired samples (Table 1).

A limitation of our study are the small amounts of blood drawn for discarding, however 0.5 mL equals multiple fillings of the cannula and should therefore secure elimination of administered saline and heparin. The low blood volume both for discarding as well as for PK-sampling, totally 1 mL, were used to minimize the blood loss for the patients and was sufficient for several HPLC analysis (Table 2). Using a radioactive substance as GFR-marker would potentially reduce the probability of marker remnants^30^. However, the fact that iohexol is a non-radioactive substance is important due to safety by repeated use in CKD children.

**Table 2:** Three step procedure.

Step 1: Pre-injection preparations	Step 2: Injection of iohexol	Step 3: Blood sample extractions post-injection
* Intravenous accesses were established in both arms * Before any injection of iohexol, a 3 mL blood sample was drawn from the cannula for biomarker measurements and to exclude any interferences with the marker	* Injection of iohexol was performed * After injection of iohexol, the intravenous access was flushed with 15 mL saline in the main entrance, 5 mL in the upper access, and thereafter 10 mL in the main entrance	* Venous blood samples (0.5 mL) were collected at 2, 3, 3.5 and 4 h after iohexol injection * Prior to drawing each blood sample, 0.5 mL blood was drawn and discarded to ensure accurate samples * After each blood sampling, the access was flushed with 1 mL isotonic saline followed by 0.2 mL heparin 100 IE/mL

The findings in our study have significant clinical value. The potential benefit of using a minimal intravenous access procedure and fewer blood samples paying attention to sufficient saline flushing are of great value in patient groups consisting of children who have difficult venous access and are subject to regular testing and treatment. In such cases a single intravenous access method may be an alternative. However, our study highlights the importance of awareness of the risk of falsely low iGFR, especially in high risk patients in need of GFR-dependent dosing of drugs as well in studies of GFR-methodology^19–22^.

In summary, the most reliable method to measure iohexol concentration and iGFR requires two venous accesses, with blood sampling from the contralateral arm. With a single intravenous access method when blood is collected from the same cannula as the iohexol injection there is still a risk of marker contamination and underestimation of GFR, despite thorough saline flushing, In cases where there is difficulty in establishing two intravenous accesses, blood samples from the injection site may be an clinical acceptable alternative provided awareness of the limitations of this method.

## Methods

### Patients

Thirty-one children with chronic kidney disease (CKD) at Haukeland University Hospital were included in the study and had complete data sets for analysis. One additional patient was initially included, but due to problems with intravenous access, this patient was excluded from the data analyses. The median age of the 31 children was 10.5 years (range 6–17 years), with median height 142 cm (range 114–177 cm) and median weight was 41 kg (19–85 kg). Written informed consent by parents/legal guardians was obtained from for all included children as well as from the included patients > 16 years of age. The study was approved by the Western Norway Regional Committee for Medical and Health Research Ethics (REK2009/741) and was in accordance with the Helsinki Declaration. The study was registered at ClinicalTrials.gov with the identifier NCT01092260, and other sub-studies on intravenous GFR methodology from the study have been published elsewhere^24,25^.

### GFR measurements

The GFR procedure was performed in three steps (Table 2). Following the first blood sample extraction, iohexol (Omnipaque 300 mg iodine/mL (GE Healthcare)) was injected. The doses of Omnipaque were calibrated according to the patient’s body weight: 10–20 kg: 2 mL; 20–30 kg: 3 mL; 30–40 kg: 4 mL; ≥ 40 kg: 5 mL. The syringe used in the injection was weighed before and after injection to calculate exact amount of Omnipaque injected^24^. After administration of iohexol, the intravenous access was flushed with a total of 30 mL saline (Table 2).

Timed blood samples of 0.5 mL were drawn from the injection access and a second cannula in the contralateral arm. Parallel samples were drawn 2, 3, 3.5 and 4 h after the injection of iohexol. Blood discarding (0.5 mL) preceded and saline flushing (1 mL) followed each blood sampling. After the saline injection 0.2 mL Heparin 100 IE/mL was administered in the veneflon.

The blood was centrifuged at 1000–1300 G for 10 min and then stored at −20 °C until analysis. Analysis of the iohexol concentration was performed by the Laboratory of Clinical Biochemistry at Haukeland University Hospital. A high performance liquid chromatography (HPLC) was used to determine the iohexol plasma concentration in the samples. The area under the largest iohexol peak was used to calculate the concentration of iohexol by comparing it to an internal calibration curve. Using this method, the coefficient of variation was 4.1% at 10 mg/L, 3.8% at 25–290 mg/L and 3.3% at > 290 mg/L.

The GFR values were calculated using Fleming’s single-point formula^23,24^ for all four time points and Jødal–Brøchner-Mortensen’s (JBM) multipoint formula^25–27^ for both two and four time points. This was done separately for the ipsilateral and contralateral cannulae.

### Statistical analysis

Agreement between GFR values from the ipsilateral and contralateral cannulae was evaluated using Bland–Altman plots^31^ (Figs. 2 and 3, Table 1). We report the bias—i.e., the mean difference in GFR based on data from the ipsilateral and contralateral arms—the 95% confidence intervals for the bias and the limits of agreement. Systematic differences in GFR values between the two arms/cannulae were tested using the Wilcoxon signed rank test, which tests for symmetry around zero. R version 4.0.4 (https://www.R-project.org/) was used for all statistical analyses and figure preparation.
